# Documentation of plant diversity of Southeast Asia: the new role of Belt and Road Initiative

**DOI:** 10.3897/phytokeys.138.49861

**Published:** 2020-01-10

**Authors:** Mung Htoi Aung, De Zhu Li, Yun Hong Tan, Nian He Xia, Rui Chuan Quan, Xiao Hua Jin

**Affiliations:** 1 Southeast Asia Biodiversity Research Institute, Chinese Academy of Sciences, Yezin, Nay Pyi Taw 05282, Myanmar; 2 State Key Laboratory of Systematic and Evolutionary Botany, Institute of Botany, Chinese Academy of Sciences, Beijing 100093, China; 3 The Germplasm Bank of Wild Species, Kunming Institute of Botany, Chinese Academy of Sciences, Kunming, Yunnan 650201, China; 4 Center for Integrative Conservation, Xishuangbanna Tropical Botanical Garden, Chinese Academy of Sciences, Mengla, Yunnan 666303, China; 5 Key Laboratory of Plant Resources Conservation and Sustainable Utilization/Guangdong Provincial Key Laboratory of Digital Botanical Garden, South China Botanical Garden, Chinese Academy of Sciences, Guangdong 510656, China

## Abstract

Editorial of special issue of plant diversity in southeastern Asia.

Myers et al. (2000) identified 25 global biodiversity hotspots, four of these hotspots, namely Indo-Burma, Sundaland, Wallacea and Phillipines, being in Southeast (SE) Asia. Biodiversity in SE Asia is under various threats (Schipper et al. 2008, Hughes 2017, Aung et al. 2020). It was estimated that SE Asia has become one of the areas with the highest deforestation rates in the world and, if the deforestation rates were to continue at the present levels (0.3% loss per year), SE Asia will lose almost three-quarters of its original forest by the next century 2100, resulting in loss of its biodiversity by up to 42% (Sodhi et al. 2004). The support of international cooperation and research efforts from regional and international expertise for biodiversity research are urgently needed in SE Asia (Sodhi et al. 2004, Hughes 2017).

The Belt and Road Initiative (BRI) is a long term programme to connect the world with the aim of increasing trade and economic growth and accerelating regional integration. BRI extends into global biodiversity conservation. SE Asia is one of the BRI regions. In order to conserve high biodiversity and promote sustainable development in SE Asia, the Southeast Asian Biodiversity Research Institute (SEABRI) was established by the Chinese Academy of Sciences (CAS), in Nay Pyi Taw in 2015 (mistakenly printed as '2014' in Jin et al. 2018) in cooperation with the Forest Research Institute under the auspices of the Ministry of Environmental Conservation and Forestry of Myanmar. With financial and personnel support from CAS, SEABRI actively participates in conservation actions by undertaking local awareness of biodiversity and public education training, awarding scholarships for young scientists and supporting regional capacity building.

The documentation of the rich biodiversity in SE Asia is the very first step towards understanding and conservation of biodiversity (Cai et al. 2019). The first special issue of the documentation of plant diversity in SE Asia by SEABRI was published in Phytokeys in 2018 (Jin et al. 2018). This successive special issue represents new contributions by SEABRI to promote our understanding of the biodiversity and conservation in the region. Seventeen articles have been contributed mainly by young botanists from Southeast Asian countries with support from SEABRI. Most articles in the issue involve the documentation of taxonomic discovery by a cooperation team of SEABRI and local botanists and includes new taxa and new records from botanical surveys in the region. They include three new fossil records of *Equisetum* (Equisetaceae) from the Neogene of South-Western China and Northern Vietnam, a new genus and species of temperate bamboo (Poaceae, Bambusoideae) from Central-Southern Vietnam and a new species of paleotropical bamboo genus *Schizostachyum* (Poaceae, Bambusoideae) from Vietnam, a new species and two new records of *Goniothalamus* (Annonaceae) from Laos and studies of *Begonia* (Begoniaceae) from Laos and Myanmar, a new species of *Bulbophyllum* (Orchidaceae) from Indonesia, two new species of *Alseodaphnopsis* (Lauraceae) from South-Western China and Northern Myanmar, a new species of *Ainsliaea* (Asteraceae) from near the border of Myanmar and China, new species of *Colocasia* (Araceae), *Ophiorrhiza* (Rubiaceae), *Blumea* (Asteraceae) and *Zingiber* (Zingiberaceae) from Myanmar and taxonomic studies on *Amomum* (Zingiberaceae) from Myanmar, an annotated checklist of Orchidaceae and notes on *Gastrochillus* (Orchidaceae) from Myanmar and a new species and two new combinations of *Monolophus* (Zingiberaceae) from Indo-Burma. All these studies were financially supported by the CAS.
